# Impact of climatic factors on water quality parameters in tilapia broodfish ponds and predictive modeling of pond water temperature with ARIMAX

**DOI:** 10.1016/j.heliyon.2024.e37717

**Published:** 2024-09-11

**Authors:** Mohammad Abu Baker Siddique, Balaram Mahalder, Mohammad Mahfujul Haque, A. K. Shakur Ahammad

**Affiliations:** a Department of Fisheries Biology and Genetics, Faculty of Fisheries, Bangladesh Agricultural University, Mymensingh, Bangladesh; b Department of Aquaculture, Faculty of Fisheries, Bangladesh Agricultural University, Mymensingh, Bangladesh

**Keywords:** Climatic factors, ARIMAX, Modeling, forecasting, Broodfish, Aquaculture, Policies, Bangladesh

## Abstract

Climate change represents a considerable threat to aquatic ecosystems, potentially affecting various water quality parameters.The study aims to assess the impacts climatic factors on the water quality parameters in tilapia broodfish pond and forecasting of water temperature in a tilapia broodfish pond using the ARIMAX model. Daily longitudinal time series data on water quality parameters were collected from the pond, while monthly climatic data were obtained from the Bangladesh Meteorological Department. Water temperature exhibited seasonal variation, peaking at 31.23 °C in October and dropping to 20.8 °C in December. pH levels ranged from 7.36 to 10.32, with the lowest recorded in December and the highest in August. Dissolved oxygen levels varied from 7.09 mg/L to 10.65 mg/L, with the lowest in September and the highest in January. Ammonia levels were highest in February at 0.33 mg/L. Water transparency ranged from 15.37 to 28 inches, with the highest in January and the lowest in June. Climatic factors significantly influenced these variations, as specified by Canonical correlation analysis (CCA). The best-fitting model, ARIMAX (1, 0, 1), indicated a fluctuating trend influenced by important exogenous factors like air temperature and solar intensity. By the end January 2025, the water temperature is expected to rise to 27.93 °C. This is a noticeable increase started from November to January. These higher temperatures may improve tilapia broodfish growth and development earlier. But the temperatures are predicted to drop started from February to March, which could negatively affect tilapia growth and development. A clear seasonal fluctuating pattern is exhibited in the future. These findings provide important insights for researchers, policymakers, academics, and those involved in tilapia farming. By considering air temperature and solar intensity in planning, stakeholders can better anticipate future pond conditions. Developing adaptive management strategies to reduce negative impacts and make the most of favorable conditions will be essential for sustainable tilapia production in the context of climate change.

## 1 Introduction

Broodfish ponds are a critical component of aquaculture, significantly contributing to the global aqua food supply [1]. The success of these ponds relies on several environmental factors, with a central focus on water quality parameters and water temperature, both of which are intricately linked to climatic conditions stated by a previous study [2]. Climate change, largely caused by human activities like burning fossil fuels, deforestation, and industrialization, has led to enduring changes in the Earth's climate [3]. Consequently, it has ushered in profound impacts on aquatic ecosystems, including rising temperatures that threaten fish species and other aquatic life [4]. These changes extend to shifts in temperature and precipitation patterns, disrupting the delicate equilibrium of dissolved oxygen, pH levels, and ammonia concentrations. Understanding the complex interaction between climatic factors and water quality parameters in ponds is crucial for maximizing production and maintaining the health of aquatic organisms [5,6].

The water quality of broodfish ponds is significantly affected by climatic factors, including air temperature, humidity, rainfall, and solar intensity, which are crucial in preserving the balance of these aquatic environments [[7], [8], [9]]. The relationship between climatic factors and water quality parameters is complex and critical.The increase in air temperature can lead to elevated water temperature, which, in turn, may lower dissolved oxygen levels due to the reduced oxygen-holding capacity of warmer water [[10], [11], [12]]. Fluctuations in water temperature can pose stress or endanger aquatic organisms, and these temperature shifts may further impact pond pH levels, often resulting in increased acidity that threatens aquatic life according to several studies [5,9]. Conversely, lower temperatures may result in alkaline conditions [13]. Air temperature can also influence ammonia levels, as higher temperatures can accelerate ammonia production, which can harm aquatic organisms [14]. Humidity, though indirectly affecting water quality, shapes evaporation and precipitation rates [15,16]. High humidity reduces evaporation, causing water temperature to rise due to reduced cooling [17,18]. Conversely, low humidity leads to increased evaporation, potentially lowering water temperature. Humidity also affects dissolved oxygen by impacting photosynthesis rates in aquatic plants [19]. High humidity restricts sunlight penetration, reducing photosynthesis and, subsequently, dissolved oxygen levels [20]. In contrast, low humidity enhances sunlight penetration, elevating dissolved oxygen. pH levels are influenced, with high humidity increasing CO_2_ levels in the air, which can dissolve into the water, lowering pH [19,21]. High humidity can promote algae growth, consuming ammonia. In contrast, low humidity may lead to higher ammonia concentrations. Rainfall can have contrasting impacts on water quality. It can cool the water, benefiting organisms preferring lower temperatures, and increase dissolved oxygen through aeration [22,23]. However, heavy rainfall may introduce pollutants and nutrients, elevating ammonia and nutrient levels, potentially leading to algal blooms. Rainfall can also impact pH, with acid rain from pollutants lowering pH and threatening pH-sensitive aquatic organisms [[24], [25], [26]]. Solar intensity affects water quality by heating the water, increasing temperature and influencing biological processes [[27], [28], [29]]. This can lead to higher dissolved oxygen levels through enhanced photosynthesis, but it can also raise respiration and decomposition rates, potentially lowering dissolved oxygen [10]. Solar intensity indirectly influences pH and ammonia by affecting biological processes like photosynthesis and respiration [9]. High solar intensity can reduce CO2 levels, elevating pH, while also releasing ammonia, potentially increasing ammonia levels [30].

In light of the rising global temperatures, it becomes decisive to forecast the future of broodfish production to ensure sustainable aquaculture practices. This contributes significantly to the formulation of aquaculture management plans that can meet the increasing demands of the growing population [[31], [32], [33]]. Predictive and simulation techniques play anessential role as they provide valuable insights into potential future scenarios. The ARIMA model, well-known for its capability to analyze time series data and generate precise forecasts, is a valuable tool in many disciplines [24,[34], [35], [36], [37]]. Acknowledging the complex nature of changes in water quality parameters, where shifts in one can influence others, this study highlights the importance of accounting for these external factors to achieve accurate water temperature predictions [1,10,38].

The changes in water quality characteristics are often hidden within aquatic ecosystems, making it challenging for farmers to detect them until they reach critical levels, leading to numerous adverse effects on fish production and financial hardships for farmers [26,39,40]. Consequently, comprehending the intricate connection between climatic factors and water quality parameters in Tilapia broodfish ponds is of immense importance for several reasons. This understanding is essential for ensuring the health and growth of fish, effectively managing water quality to identify potential risks, and mitigating environmental shifts that could harm aquatic life [41]. It also contributes to the promotion of environmentally sustainable aquaculture by preserving the health of aquatic ecosystems. While tilapia is relatively resilient and help maintain a clean pond environment, but extreme weather conditions can still harm them.

Therefore, the comprehensive presentation and contextual analysis provided herein underline the complex relationship among the water quality parameters. There remains a notable gap in the literature concerning broader-scale studies incorporating multiple climatic factors and water quality parameters simultaneously. Furthermore, it has been observed that water temperature is significantly influenced by climatic factors. It is evident that climatic factors directly and indirectly influence on the pond water quality parameters. If one changes happen and make changes others in the pond water ecosystem and ultimately make abrupt impact on the broodfish growth and development. As the important factor in the pond water condition, determine the future phenomena of pond water temperature through modeling and forecasting will be essential for planning and management for sustainable broodfish production. Water temperature follows seasonal pattern by influencing several exogenous factors in the pond water system. Hence, there arises a necessity for ARIMA modeling and forecasting, augmented by ARIMAX, which incorporates exogenous factors such as air temperature and solar intensity leading for making seasonal pattern. Remarkably, such an approach has not been widely explored in the fisheries sector. Analyzing the impact of climatic factors on water quality parameters in Tilapia broodfish ponds and utilizing ARIMAX modeling for predictive water temperature assessments poses a complex issue with significant implications. This study stands as a pioneering effort, bridging a crucial gap in fish hatchery practices and delving into the intricate interplay between climate change and water quality. By employing advanced modeling techniques, this research can address critical challenges within the aquaculture sector, particularly in Bangladesh. The present study aims to fill this gap by employing the ARIMA model in conjunction with external factors (ARIMAX) to anticipate water temperature in Tilapia broodfish ponds. It is anticipated that these predictions will furnish invaluable insights for fish farmers, policymakers, and stakeholders within the aquaculture industry.

## 2 Materials and methods

### 2.1 Study area

The study was carried out in a Tilapia broodfish pond situated at Dhala, Trishal, Mymensingh, Bangladesh, about 37 km south of Bangladesh Agricultural University in Mymensingh, Bangladesh (Fig. S1). A longitudinal study was conducted from February 2021 to January 2022. The study area is characterized by favorable topographic conditions. It is predominantly a flat, lowland region with clay soil. The local population primarily engages in fish farming and various agricultural activities. Historically, the community has a strong tradition of fish seed production and aquaculture. In terms of morphometric parameters, the experimental pond covered a surface area of 35 decimals, equivalent to 15,246 square feet. The average depth of the pond was about 4 feet, with a maximum depth of 4.5 feet.

### 2.2 Collection of climatic variables and water quality parameters in tilapia broodfish pond

Monthly data on climatic variables, including air temperature, humidity, rainfall, and solar intensity, were sourced from the Bangladesh Meteorological Department. Daily longitudinal time series data on essential water quality parameters—such as water temperature, dissolved oxygen (DO), pH, ammonia, and water transparency—were collected using various tools, including a SMART Sensor temperature meter (SMART Sensor AR 867), a Lutron DO-5509 DO meter, a compact pH meter (pH-107), an ammonia test kit, and a Secchi disk for measuring water transparency.All the data were documented in the excel file initially and then compiled and analyzed according to key aspects and indicators.

### 2.3 Correlation between climatic factors and water quality parameters

Canonical Correlation Analysis (CCA) was employed to explore the relationship between climatic factors and water quality parameters. In the initial step of CCA, one or more canonical functions are generated. Each function consists of a pair of variables: one serving as the explanatory or independent variable, which includes climatic factors such as air temperature, humidity, rainfall, solar intensity, and wind speed; the other serving as the response or dependent variable, which includes water quality parameters like dissolved oxygen (DO), pH, water temperature, and water transparency. The first canonical function extracted is designed to capture the maximum variance between these two sets of variables, reflecting the strongest possible inter-correlation. Additional functions are derived to account for any remaining variance. The interpretation of canonical functions is generally based on two main criteria: the statistical significance of the function and the strength of the canonical correlation. In this study, the explanatory variables were denoted as Y variables in the c vector (c_1_, c_2_, …, c_Y_), while the response variables were denoted as X variables in the k vector (k_1_, k_2_, …, k_X_). Thus, observations j can be characterized using these vectors.

$$\left[\begin{array}{c} c_{1}\\ k_{1} \end{array}\right]..\ldots .\left[\begin{array}{c} c_{n}\\ k_{n} \end{array}\right]$$

with a partitioned sample

$$\bar{x}=\left[\begin{array}{c} {\bar{x}}_{C}\\ {\bar{x}}_{k} \end{array}\right]$$

and a partitioned sample variance

$$S=\left[\begin{array}{cc} S_{cc} & s_{kc}\\ S_{kc} & S_{kk} \end{array}\right]$$

were,

$$S_{cc}=\frac{1}{n-1}\sum_{j=1}^{n}\left(c_{j}-{\bar{x}}_{c}\right){\left(c_{j}-{\bar{x}}_{c}\right)}^{\prime }$$

$$S_{kk}=\frac{1}{n-1}\sum_{j=1}^{n}\left(k_{j}-{\bar{x}}_{k}\right){\left(k_{j}-{\bar{x}}_{k}\right)}^{\prime }$$

and

$$S_{ck}=\frac{1}{n-1}\sum_{j=1}^{n}\left(c_{j}-{\bar{x}}_{c}\right){\left(k_{j}-{\bar{x}}_{k}\right)}^{\prime }$$

The variance-covariance matrices S_cc_ and S_kk_ represent the variances and covariances within the groups of explanatory and response variables, respectively. In contrast, the matrices S_kc_ and S_ck_ represent the covariances between variables from different groups. When explanatory and response variables are linearly combined, they create canonical functions denoted as s = (s_1_,s_2_, …,s_Y_) and p = (p_1_,p_2_, …,p_X_). These vectors are composed of climatic variables (explanatory variables) in canonical variates and are formed by a linear combination of the c vector with canonical coefficients, represented by vector a, expressed as s = a′c. Vector p includes growth parameters (response variables) in canonical variates and is derived from a linear combination of the k vector with canonical coefficients, represented by vector b, shown as p = b′k. The canonical correlation coefficients a and b are estimated using the variance-covariance matrices of the original sets of explanatory and response variables, as detailed in Equations (1), (2).

(1) $$S_{cc}^{-1}S_{ck}S_{kk}^{-1}S_{kc}a_{i}=\lambda_{i}a_{i}$$

(2) $$S_{cc}^{-1}S_{ck}S_{kk}^{-1}S_{kc}b_{i}=\lambda_{i}b_{i}$$

Pairs of canonical variables, denoted as (s_i_, p_i_), are generated through linear combinations such as s_i_ = a_i_′c and p_i_ = b_i_′k. These pairs show stronger correlations and greater independence compared to subsequent pairs of canonical variables, like (s_i+1_, p_i+1_), which are produced using equations such as s_i+1_ = a_i+1_′c and p_i+1_ = b_i+1_′k. This process continues until the final pairs, (s_Y_, p_Y_), are obtained. Canonical Correlation Analysis (CCA) specifically aims to maximize the correlation between the first pair of canonical variables, such as s_1_ = a_1_′c and p_1_ = b_1_′k. The second pair, s_2_ = a_2_′c and p_2_ = b_2_′k, achieves the next highest correlation and remains uncorrelated with the initial pair (s_1_, p_1_).

### 2.4 Predictive modeling of pond water temperature using ARIMAX

In this study, we utilized the ARIMAX model (Autoregressive Integrated Moving Average with Exogenous Variables), a time series forecasting technique that expands upon the ARIMA model by including external variables. This approach follows the Box-Jenkins methodology [42]. This approach relies on the analysis of longitudinal time series multifactorial data, proving to be a powerful tool in various domains [[43], [44], [45]]. The ARIMAX model, a statistical tool, was used to forecast future values of a dependent variable by considering both independent variables and the historical values of the dependent variable. These models are proficient at identifying linear relationships between previous and current data points and effectively managing any errors or residuals present in the data. The process of ARIMAX modeling and forecasting can be outlined as follows:

#### 2.4.1 Data preprocessing

After gathering data from experimental and secondary sources, the first step was to preprocess the data. This involved examining it for missing values and addressing these gaps through mean imputation, median imputation, and removing rows with missing data. Additionally, outliers were managed by either removal or transforming them to a fixed value. Necessary data transformations were also performed as needed.

#### 2.4.2 Model identification

The ARIMA modeling process involved analyzing the autocorrelation function (ACF) and partial autocorrelation function (PACF) of the time series data to identify potential models with similar orders. Once the potential models were identified, the next step was to estimate the parameters, focusing on three key aspects: the autoregressive order AR(p), the integration order I(d), and the moving average order MA(q). The Bayesian Information Criterion (BIC) was used to select the best values for these parameters.

The MA(q) model specifically refers to the moving average component of the ARIMA model, which is defined as:

(3) $$X_{t}=\mu +\varepsilon_{t}+\theta_{1}\varepsilon_{t}-1+\theta_{2}\varepsilon_{t-2}+\ldots +\theta_{q}\varepsilon_{t-q}$$

Here, X_t_ represents the observed value at time t, μ denotes the mean of the time series, ε_t_ is the white noise error term at time t, and θ_1_, θ_2_, …,θ_q_ are the parameters that need to be estimated.

The AR(p) model, which represents the autoregressive part of an ARIMA model, is formulated as follows:

(4) $$X_{t}=\phi_{1}X_{t-1}+\phi_{2}X_{t-2}+\ldots +\phi_{p}X_{t-p}+\varepsilon_{t}$$

Here, X_t_ is the observed value at time t, ϕ_1_, ϕ_2_, …,ϕ_p_ are the autoregressive coefficients, and ε_t_ is the white noise error term at time t.

For both models, the white noise error term ε_t_ is assumed to have a mean of zero and a constant variance. Accurately identifying and estimating these elements is essential for building a strong ARIMA model that can effectively capture the underlying patterns in time series data.

#### 2.4.3 Estimation of parameters

In the ARIMA modeling process, the parameters for the selected initial models were identified. Generally, parameter estimation for the ARIMA model was carried out using the nonlinear least-squares method, as mentioned in previous studies [46]. Various metrics were used for parameter estimation, including root mean square error (RMSE), mean absolute percentage error (MAPE), maximum absolute percentage error (MaxAPE), mean absolute error (MAE), maximum absolute error (MaxAE), and the normalized Bayesian Information Criterion (BIC). These metrics were considered crucial in evaluating the accuracy of the ARIMA model. The time series longitudinal data was carefully examined for stationarity, and appropriate differencing was applied as needed during the calibration of the ARIMA model. The parameters, such as AR(p) and MA(q), were determined using autocorrelation and partial autocorrelation functions. The model was then fitted to the training dataset, and diagnostic checks on the residuals were conducted to ensure the model's adequacy. Next, the model was tested on unseen data to validate its performance, with its predictive accuracy evaluated using metrics like RMSE, MAE, MSE, MAPE, and BIC. The process involved iterative refinement, including adjustments to parameter values, to develop a well-calibrated and validated ARIMA model, suitable for making reliable predictions on new data. The model with the best forecasting performance is the one with the lowest error criterion value [47].

(5) $$\text{MSE}=\frac{1}{n}\sum_{t=1}^{n}e_{t}^{2}\text{,}$$

(6) $$\text{RMSE}=\sqrt{\frac{1}{n}\sum_{t=1}^{n}e_{t}^{2}}\text{,}$$

(7) $$\text{MAE}=\frac{1}{n}\sum_{t=1}^{n}⃒e_{t}⃒\text{,}$$

(8) $$\text{MAPE}=\frac{100\%}{n}\sum_{t=1}^{n}\left|\frac{e_{t}}{y_{t}}\right|$$

Here *e*_*t*_ represents the error term, $y_{t}$ is the observed value, and $\tilde{y_{t}}$ is the forecasted value, and $e_{t}$ = $y_{t}-\tilde{y_{t}}$,

Bayesian Information Criteria (BIC) introduced in 1978, is calculated as

(9) $$\text{BIC}=T^{\prime }\;\log\left(\sigma^{2}\right)+\left(p+q+1\right)\;{\text{logT}}^{\prime }$$

Here, $\sigma^{2}$ represents the mean square error, and T'denotes the number of observations. The model with the smallest BIC value is considered the most optimal [46].

In addition, the estimation of the parameters was also evidenced for water temperature based on Coef, SE Coef, T-value, and P-value. The formula for the coefficient (Coef) in a linear regression model was calculated using the following matrix equation: b = (X'X)^-1 X'y, where X was the design matrix, y was the response vector, and X′ was the transpose of the design matrix. The standard error of the coefficient (SE Coef) was calculated using the following formula: SE Coef = sqrt(MSE ∗ (X'X)^-1), where MSE was the mean square error. The T-value for a coefficient was calculated as: T-value = Coef/SE Coef. The P-value for a coefficient could be obtained from the t-distribution table using the degrees of freedom and T-value.

#### 2.4.4 Diagnostic test of residuals

The diagnostic process began with an examination of the autocorrelation function (ACF) and partial autocorrelation function (PACF) of the residuals to check for adherence to white noise patterns. If the residuals did not exhibit white noise characteristics, additional checks were conducted to evaluate their randomness and adherence to a normal distribution. Both the ACF and PACF remained within the specified thresholds, confirming the accuracy of the ARIMA model. To further validate the model's adequacy, a verification process was carried out, which included a detailed analysis of the normal probability plot and histogram of the residuals. These visual tools helped determine whether the residuals were normally distributed. The normal probability plot showed that data points were closely aligned with a straight line, suggesting approximate normality, while the histogram displayed a bell-shaped curve, indicating a normal distribution. This comprehensive analysis was essential in confirming that the residuals met the assumption of normality, with graphical representations clearly illustrating the distribution characteristics. The selection of the most suitable ARIMA and ARIMAX models was based on specific analyses and contextual factors, using criteria such as the lowest values of RMSE, MAPE, MaxAPE, MAE, MaxAE, and normalized BIC to identify the best-performing models.

#### 2.4.5 Cross correlation

The analysis involved using the cross-correlation function (CCF) to examine the relationship between air temperature and solar intensity as independent variables and water temperature as the dependent variable. The climate variables were considered to be independent and were pre-whitened using previously fitted ARIMA models. Pre-whitening is a crucial technique used to determine the time lag of the independent variable that affects the dependent variable. This method is widely used to study various geophysical time series variables [48]. After pre-whitening, the selected climate variables from the previous step were included as covariates in the ARIMAX model. The CCF was then used to measure the level of similarity between the two-time series at different lags. The lag represents the time difference between the two series, and the CCF values range from −1 to 1. A value of −1 indicates a perfect negative correlation, 0 indicates no correlation, and 1 indicates a perfect positive correlation. A positive CCF value suggests that as one time series increases, the other also increases, while a negative value indicates that as one time series increases, the other decreases.

#### 2.4.6 Forecasting

The final step in ARIMA modeling involved generating forecasts and evaluating the model's accuracy by comparing it with other models using metrics such as RMSE, MAPE, MaxAPE, MAE, MaxAE, and normalized BIC values. Forecasts for the sample period are used to assess the model's reliability, while forecasts beyond the sample period provide more accurate predictions for policymaking and other practical applications.

In the ARIMA (p, d, q) model, the future value of a variable is calculated as a linear combination of its past values and previous errors, represented as follows:

(10) $$\Delta^{d}+Y_{t}=\emptyset_{0}+\Delta^{d}Y_{t}=\emptyset_{0}+\emptyset_{1}\Delta^{d}Y_{t-1}+\emptyset_{2}\Delta^{d}Y_{t-2}+\ldots \ldots +\emptyset_{p}\Delta^{d}Y_{t-p}+\varepsilon_{t}+\theta_{1}\varepsilon_{t-1}+\theta_{2}\varepsilon_{t-2}+\ldots ..+\theta_{q}\varepsilon_{t-q}$$

Where, Y_t_ denotes the actual value at time t, and ε represents the random error at that same point in time. The parameter d specifies the number of differencing transformations needed for the time series to become stationary. θ_t_ and θ_j_ are coefficients, with p and q being integers commonly referred to as autoregressive and moving average parameters, respectively. The autoregressive component accounts for the influence of previous observations, while the moving average component reflects the effect of past forecast errors. Together with the constant term (ϕ_0_), these elements are vital for modeling time series data using the ARIMA approach.

#### 2.4.7 Simulation

Simulation was employed as a forecasting method when a mathematical model proved inadequate or overly complex for analytical solutions. In the context of our ARIMAX modeling and forecasting, we used simulation to account for uncertainty and variability in our predictions, thereby improving their reliability for decision-making. This approach proved especially valuable when multiple sources of uncertainty influenced future outcomes, allowing us to quantify and manage associated risks. To apply simulation in ARIMAX modeling, we obtained forecasted values and then used them to generate future scenarios. This involved selecting random numbers from appropriate sources to determine the sequence of values for the variable of interest, and subsequently fitting a standard mathematical function to these simulated values. This process enabled us to explore potential outcomes and assess the likelihood of different scenarios, providing us with crucial insights for complex and uncertain situations. The simulation patterns provide concrete evidence and substantiate the forecasted values of water temperature using ARIMAX.

### 2.5 Statistical analysis

The data was first organized, combined, and structured using according to various parameters in Microsoft Excel 2016. Descriptive statistics for the time series longitudinal data on climatic factors and water quality parameters were calculated using Minitab 2019 software. Following this, the findings were sorted into different categories based on specific criteria. For Canonical Correlation Analysis (CCA), the software used was SPSS version 2023. ARIMA modeling was performed using SPSS (version 2023) and Minitab (version 2019) to model and analyze time series data with practical implications. Furthermore, forecasting the water temperature, considering influential exogenous factors (ARIMAX), and simulating it, were achieved using the NumXL add-ins in version 2016. The output of the analysis was then presented according to various indicators and key aspects.

## 3 Results

### 3.1 Analysis of climatic factors and water quality parametersin tilapia broodfish pond

Throughout the duration of the study, fluctuations in various climatic factors and water quality parameters were observed. In terms of climatic factors, air temperature ranged from 18.93 °C to 30.12 °C, humidity varied between 75 % and 85.8 %, rainfall fluctuated from 0 mm to 485 mm, solar intensity ranged from 3.02 h to 7.9 h, and wind speed ranged from 0.338 km/h to 7.028 km/h. Regarding water quality parameters, water temperature fluctuated between 20.81 °C and 31.23 °C, dissolved oxygen (DO) levels varied from 7.09 mg/L to 10.65 mg/L, pH values ranged from 7.36 to 10.32, ammonia concentrations fluctuated from 0 mg/L to 0.333 mg/L, and water transparency varied between 15.37 cm and 27.05 cm.

### 3.2 Canonical correlation between climatic factors and water quality parameters

Table 1 presented the relationships between multiple sets of variables using Canonical Correlation Analysis (CCA). CCA allowed for the exploration of correlations between climatic variables, including air temperature, humidity, rainfall, solar intensity, and wind speed, and water quality parameters such as water temperature, dissolved oxygen (DO), pH, ammonia, and water transparency in the tilapia broodfish pond. The calculated canonical correlations between the pairs of canonical variates were found to be 0.892, 0.431, 0.272, 0.151, and 0.006 with corresponding significance probabilities.

**Table 1:** Canonical correlation between climatic factors and water quality parameters.

Canonical Correlations					
Variable	1	2	3	4	5
**Independent variables**					
Air temperature	**−0.990**	−0.025	0.110	−0.073	−0.031
Humidity	−0.067	0.006	**0.930**	0.230	0.279
Rainfall	−0.275	0.355	**0.555**	0.287	**−0.639**
Solar intensity	0.038	−0.362	**−0.719**	**0.584**	−0.093
Wind speed	**−0.546**	**0.796**	−0.013	0.151	0.212
**Dependent variable**					
Water temperature	**−0.937**	−0.314	−0.009	0.150	−0.009
DO	**0.663**	0.322	−0.080	**0.515**	**−0.431**
pH	**−0.669**	**0.657**	−0.049	−0.286	−0.191
Ammonia	−0.226	0.364	**−0.561**	**0.418**	**0.572**
Water transparency	0.156	0.069	**−0.792**	**−0.455**	−0.371
**Canonical Correlations**	**0.892**	**0.431**	**0.272**	**0.151**	**0.006**

#### 3.2.1 Features of the first canonical function

**Fig. 1:**
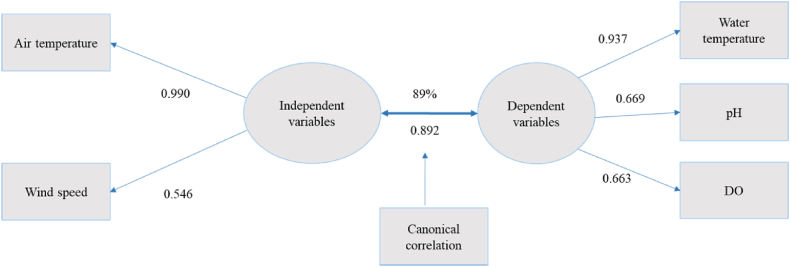
Canonical function 1 (adapted from Table 1).

The first canonical function, outlined in Table 1 and shown in Fig. 1, highlights the relationships between climatic variables (such as air temperature and wind speed) and different water quality parameters (including water temperature, pH, and dissolved oxygen) in a tilapia broodfish pond. This function exhibited a very strong correlation of 0.892, which accounts for 89 % (Fig. 1). This function provided insights into the contribution of individual variables to the observed variance. In this function, air temperature (loading of 0.990) and wind speed (loading of 0.546) collectively explained 27.2 % of the variance, while water temperature (loading of 0.937), pH (loading of 0.669), and dissolved oxygen (DO) (loading of 0.663) collectively explained 36.8 % of the variance (Fig. 1). It is important to note that canonical loadings equal to or exceeding 0.30 were considered significant for interpretation, as lower values indicated weaker correlations between the two datasets.

#### 3.2.2 Features of the second canonical function

**Fig. 2:**
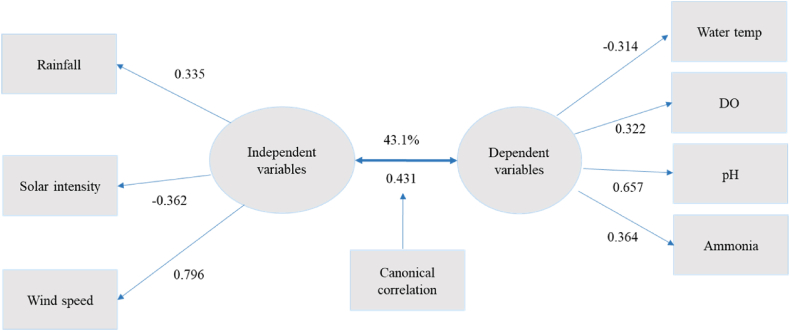
Canonical function 2 (adapted from Table 1).

Features of the second canonical function, as detailed in Table 1 and shown in Fig. 2, reveal a significant correlation of 0.431, accounting for 43.1 % of the associations between climatic conditions and water quality measures. In this function, rainfall (loading of 0.335), solar intensity (loading of −0.362), and wind speed (loading of 0.796) collectively explained 17.8 % of the variance, while water temperature(loading of −0.314), DO (loading of 0.322), pH (loading of 0.657, and ammonia (loading of 0.364) contributed to 15.4 % of the variance (Fig. 2).

#### 3.2.3 Features of the third canonical function

**Fig. 3:**
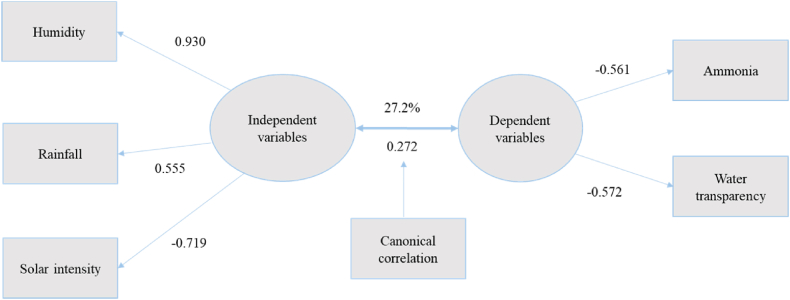
Canonical function 3 (adapted from Table 1).

Features of the third canonical function, as depicted in Table 1 and visualized in Fig. 3, there was a significant correlation of 0.272, explaining 27.2 % of the observed associations between climatic variables and water quality parameters. Within this function, humidity (loading of 0.930), rainfall (loading of 0.555) and solar intensity (loading of −0.719) was responsible for 34 % of the variance, while ammonia (loading of −0.561) and water transparency (loading of −0.572) collectively accounted for 19 % of the variance (Fig. 3).

#### 3.2.4 Features of the Fourth Canonical function

**Fig. 4:**
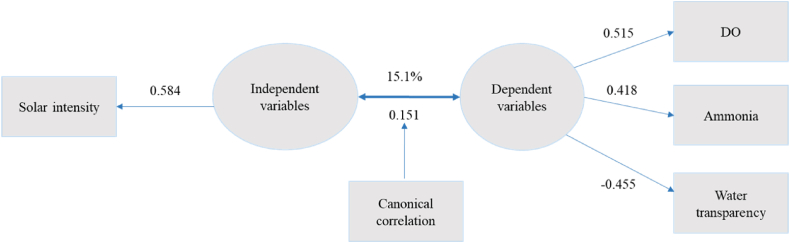
Canonical function 4 (adapted from Table 1).

Features of the fourth canonical function, as detailed in Table 1 and depicted in Fig. 4, displayed a correlation of 0.151, accounting for 15.1 % of the established links between climatic factors and water quality parameters. In this function, the variables solar intensity (loading of 0.584) contributed to 10 % of the variance, while dissolved oxygen (DO) (loading of 0.515) and ammonia (loading of 0.418), and water transparency (-0.455) collectively accounted for 15 % of the variance (Fig. 4).

### 3.3 Predictive modeling of pond water temperature with ARIMAX

**Fig. 5:**
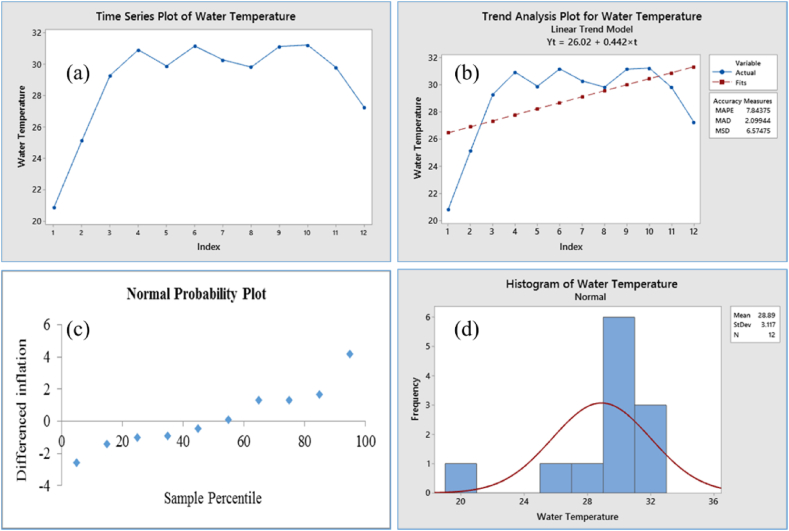
Stationary data of water temperature; (a) time series plot, (b) trend analysis plot, stationary, (c) normal probability plot of water temperature by ADF test, stationary, observations clustered within a low percentile region of the ADF test statistic's distribution, (d) normal histogram of water temperature, stationary as the data exhibits normal distribution and has a distinctive peak in the center, gradually tapering off.

**Fig. 6:**
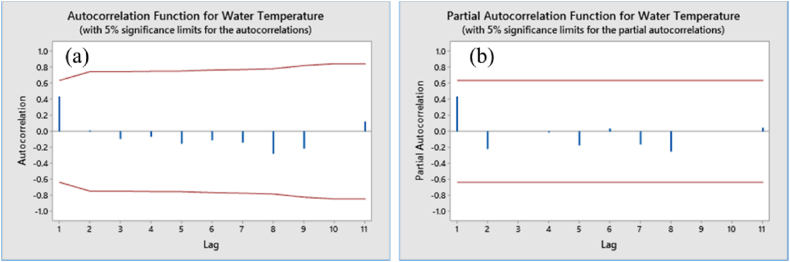
ACF & PACF of water temperature with 5 % significance limit. Showed stationary pattern as the plots indicating a rapid decline in correlations, weakening ACF associations with greater lags, and significant PACF spikes primarily at the initial lags.

The Augmented Dickey-Fuller (ADF) test applied to the time series data of pond water temperature yielded a p-value of 0.0134, which is less than the significance level of 0.05, suggesting that the data is stationary. When examining the normal probability plots for the ADF test, it was observed that the water temperature's original data points clustered within a lower percentile range of the ADF test statistic's distribution, signifying stationarity as shown in Fig. 5 (panel c). The Anderson-Darling test, applied to the normal probability plot, confirms stationarity as evidenced by a p-value of 0.005, which falls below the critical significance level of 0.05. Additional confirmation of stationarity was obtained from trend analysis and normal histograms, as evident in Fig. 5 (panels a, b, and d). The Autocorrelation Function (ACF) and Partial Autocorrelation Function (PACF) plots provided valuable insights into the longitudinal time series data. These plots demonstrated that the original data is stationary.The ACF plot displayed a rapid decline in correlations, indicating that the data points are only correlated over short periods, while the associations within the ACF decreased at longer lags, suggesting diminishing influence of past values over time. Moreover, the PACF plot showed noticeable spikes primarily at the initial lags, indicating significant partial correlations with recent lags but not with more distant observations (Fig. 6, panels a & b). This indicated that the data was inherently stationary, devoid of persistent trends or seasonality, enabling straightforward time series longitudinal data analysis without extensive transformations. The spikes occurring beyond the defined bounds were considered statistically significant, signifying correlations between observations and their respective lags. Fundamentally, the ACF graph depicted the correlations between sequential observations and their lags within the time series data, facilitating the identification of the Moving Average (MA) component's order in models such as ARIMA.

Table 2 displayed the coefficients, standard errors (SE), T-values, and P-values for the ARIMA model's final parameters. The estimates of the coefficients shed light on the strength and direction of the connections between lag terms and the water temperature variable. The size of each coefficient indicated the degree of influence, with higher values suggesting a more substantial effect of the predictor on the response variable. For example, an AR (1) coefficient of 1.197 pointed to a significant positive correlation between the data in the previous period and the current period's water temperature. Similarly, the negative coefficient estimates of −0.969 for the MA (1) term indicated a larger negative relationship between the residual error and the current period's water temperature. The standard errors served as estimations of the precision and reliability of the coefficient estimates. Smaller standard errors, such as the AR (1) estimate of 0.839 and the MA (1) estimate of 0.374, indicated more precise coefficient estimates with lower variability. To assess the statistical significance of the coefficient estimates, T-values were analyzed. Larger absolute values of these T-values indicate a higher level of significance. In the case of the AR (1) and MA (1) coefficients, higher absolute T-values of 1.43 and −2.59, respectively, indicated increased significance.The P-values associated with each coefficient estimate indicate the likelihood of encountering a T-value as extreme as the one observed, under the assumption that the null hypothesis (that the coefficient is zero) holds true. Relatively lower P-values, such as 0.249 for the AR (1) coefficient and 0.081 for the MA (1) coefficient, offer robust evidence against the null hypothesis. Taking into account the coefficients, standard errors, T-values, and P-values, the AR (1) and MA (1) model was determined to be the most suitable for the analysis.

**Table 2:** Estimation of the parameters for water temperature model based on Coef, SE Coef, T-value and P-value.

Type	Coef	SE Coef	T-Value	P-Value
**AR 1**	**1.197**	**0.839**	**1.43**	**0.249**
AR 2	0.137	0.906	0.15	0.889
AR 3	−0.147	0.836	−0.18	0.872
AR 4	−0.676	0.910	−0.74	0.512
AR 5	0.465	0.699	0.67	0.554
**MA 1**	**−0.969**	**0.374**	**−2.59**	**0.081**
MA 2	−0.54	1.40	−0.38	0.726
MA 3	−0.28	1.78	−0.16	0.886
MA 4	0.38	1.19	0.31	0.773

**Fig. 7:**
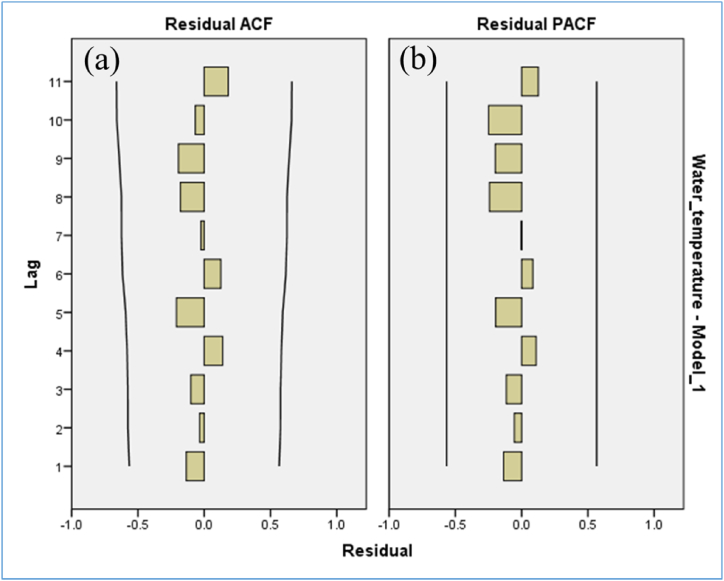
Residual ACF and PACF with normal spike distribution.

Table 3 provided a comparative analysis of different models using metrics such as RMSE, MAPE, MaxAPE, MAE, MaxAE, and normalized BIC. The ARIMA (1,0,1) model emerged as the most effective, exhibiting the lowest BIC along with comperative satisfactory values for RMSE, MAPE, MaxAPE, MAE, and MaxAE, thus proving optimal for tilapia production forecasting. Further examination of the model's residual ACF and PACF indicated normally distributed spikes. Additionally, the absence of white noise errors in the time series further confirmed that the ARIMA (1,0,1) model provided the best fit (Fig. 7a & b).

**Table 3:** Suitable model fitting for water temperature data. The model is accepted with minimum normalized BIC (∗) value were considered for the models.

Variable	Model	RMSE	MAPE	MaxAPE	MAE	MaxAE	Normalized BIC
Water temperature	ARIMA (1,0,1)	2.300	5.978	14.966	1.616	3.245	2.494∗
	ARIMA (2,0,2)	2.692	5.786	21.586	1.524	4.492	3.223
	ARIMA (3,0,3)	3.222	5.852	20.438	1.554	4.253	3.997
	ARIMA (4,0,4)	5.811	6.375	26.829	1.688	5.583	5.590

**Fig. 8:**
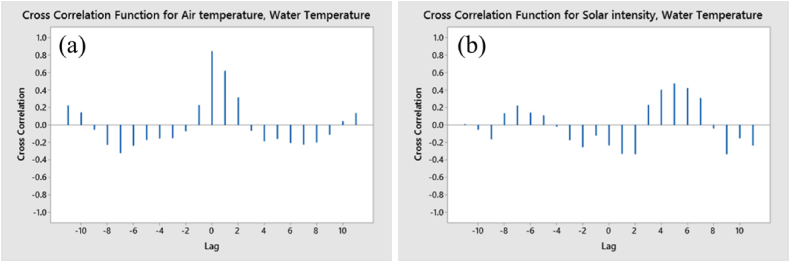
Cross correlation; (a) air temperature and water temperature and (b) solar intensity and water temperature.

The Cross-Correlation Function (CCF) served as a statistical method to assess the similarity between two time series. For our analysis, we examined pairs: air temperature versus water temperature, and solar intensity versus water temperature, as presented in Table 4 and Fig. 8 (panel a & b). The CCF quantified the correlation between these series by considering the lag, or time offset, between them. This function evaluated how effectively one series could predict the other across various lags. CCF values ranged from −1, indicating a perfect negative correlation, to 1, denoting a perfect positive correlation, with 0 reflecting no correlation at all. A positive CCF value indicated that as one-time series increased, so did the other, while a negative CCF value indicated that as one-time series increased, the other decreased. Looking at Table 4 we provided, for both pairs of time series, there were both positive and negative CCF values. There was a positive cross-correlation function (CCF) value of 0.844934 at lag 0 between air temperature and water temperature, indicating that as air temperature increased, water temperature increased as well. There were also negative CCF values at lags −11 through −2 and lags 4 through 11, indicating that as air temperature increased, water temperature decreased after a certain lag.For solar intensity and water temperature, there was a negative CCF value at lag 0 (−0.234222), indicating that as solar intensity increased, water temperature decreased. There were also positive cross-correlation function (CCF) values at lags 5 through 11, indicating that as solar intensity increased, water temperature also increased after a certain lag. Overall, the cross-correlation analysis showed how air temperature and solar intensity affected water temperature over time.

**Table 4:** Cross correlation of air temperature & water temperature and solar intensity & water temperature.

Air temperature and Water Temperature		Solar Intensity and Water Temperature	
Lag	CCF	Lag	CCF
−11	0.222196	−11	0.010135
−10	0.143471	−10	−0.053517
−9	−0.053327	−9	−0.165241
−8	−0.229414	−8	0.134987
−7	−0.322769	−7	0.220459
−6	−0.238741	−6	0.141882
−5	−0.173042	−5	0.110862
−4	−0.155182	−4	−0.020242
−3	−0.15114	−3	−0.173545
−2	−0.071981	−2	−0.254182
−1	**0.229324**	−1	**−0.1225**
0	0.844934	0	−0.234222
1	0.619471	1	−0.33217
2	0.315289	2	−0.335262
3	−0.066495	3	0.230539
4	−0.187756	4	0.403689
5	−0.160532	5	0.474457
6	−0.20793	6	0.422073
7	−0.227085	7	0.308088
8	−0.200693	8	−0.041638
9	−0.111546	9	−0.335982
10	0.044875	10	−0.152681
11	0.138072	11	−0.235989

**Fig. 9:**
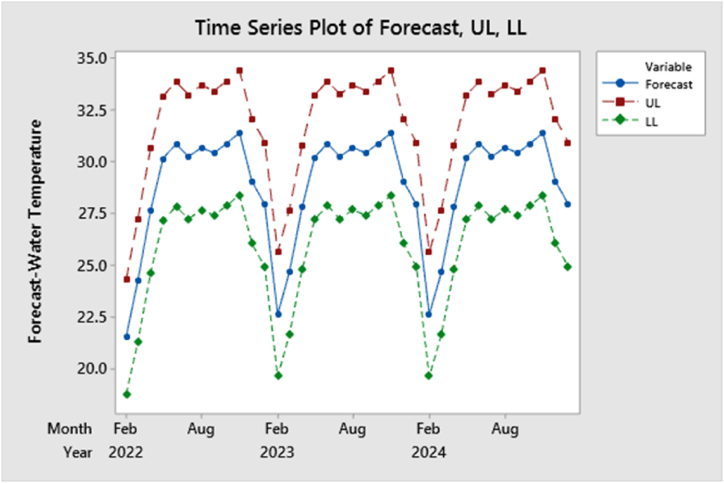
Forecasting the water temperature up to the end of January 2025 with ARIMAX.

Projected water temperatures in the broodfish pond over the next three years are outlined in Table 5 and illustrated in Fig. 9, all within the 95 % confidence intervals. Utilizing the ARIMAX (1, 0, 1) model, Fig. 9 depicts the expected trend of water temperature in the broodfish pond, showing a steady rise with periodic fluctuations. By the end of January 2025, the temperature is anticipated to reach 27.93 °C, marking a significant increase from 2021 levels. Such a temperature is deemed optimal for the growth and development of Tilapia broodfish. Conversely, the predicted water temperature decreases from February to March, which may be unfavorable for tilapia growth and development. Therefore, the seasonal fluctuations and season changes pattern might be an issue for future management.

**Table 5:** Forecasting of water temperature along with 95 % confidence interval up to the end of January 2025.

Month/Year	Water temperature (°C) Forecast	Upper confidence limit (UCL)	Lower confidence limit (LCL)
Feb 22	21.55	24.31	18.78
Mar 22	24.23	27.20	21.26
Apr 22	27.64	30.64	24.64
May 22	30.15	33.15	27.15
Jun 22	30.84	33.85	27.84
Jul 22	30.23	33.23	27.23
Aug 22	30.67	33.67	27.67
Sep 22	30.41	33.41	27.41
Oct 22	30.87	33.88	27.87
Nov 22	31.38	34.39	28.38
Dec 22	29.05	32.05	26.04
Jan 23	27.93	30.93	24.93
Feb 23	22.64	25.64	19.63
Mar 23	24.65	27.66	21.65
Apr 23	27.80	30.81	24.80
May 23	30.21	33.21	27.21
Jun 23	30.87	33.87	27.87
Jul 23	30.24	33.24	27.24
Aug 23	30.68	33.68	27.67
Sep 23	30.41	33.42	27.41
Oct 23	30.88	33.88	27.87
Nov 23	31.38	34.39	28.38
Dec 23	29.05	32.05	26.04
Jan 24	27.93	30.93	24.93
Feb 24	22.64	25.64	19.63
Mar 24	24.65	27.66	21.65
Apr 24	27.80	30.81	24.80
May 24	30.21	33.21	27.21
Jun 24	30.87	33.87	27.87
Jul 24	30.24	33.24	27.24
Aug 24	30.68	33.68	27.67
Sep 24	30.41	33.42	27.41
Oct 24	30.88	33.88	27.87
Nov 24	31.38	34.39	28.38
Dec 24	29.05	32.05	26.04
Jan 25	27.93	30.93	24.93

**Fig. 10:**
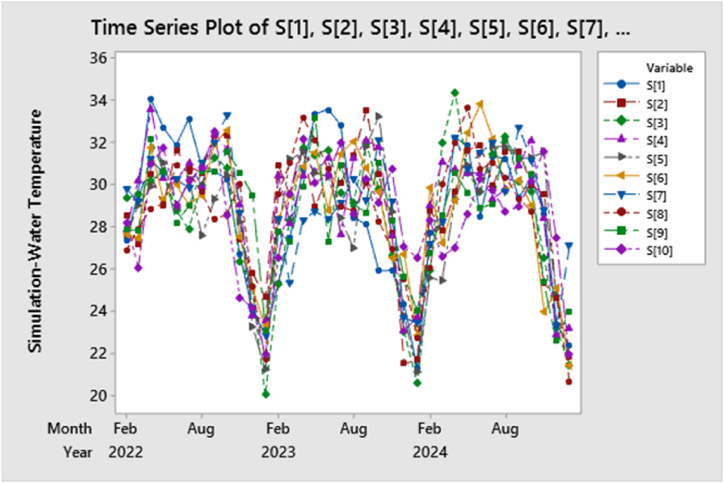
Simulation of water temperature up to the end of January 2025 with ARIMAX.

In the simulation (Table 6 & Fig. 10), water temperature in a tilapia broodfish pond over the years exhibited a clear seasonal pattern in accordance with the original longitudinal data series, strongly supporting the findings obtained from the forecasting analysis. The data revealed a gradual increase in temperature from the lower range, around 21 °C in December and January, to the higher range of approximately 34 °C in July, August, and September, characteristic of summer's warmth, before gradually declining again through autumn and winter. This cyclic temperature variation reflected the pond's responsiveness to external factors, particularly air temperature and solar intensity. These insights were of significant practical importance for us as we managed the broodfish pond and provided valuable data for our ecological conservation efforts, enhancing our understanding of the impact of seasonal temperature changes on local aquatic ecosystems and habitats.

**Table 6:** Simulation of water temperature in a tilapia broodfish pond over the years.

Step	S[1]	S[2]	S[3]	S[4]	S[5]	S[6]	S[7]	S[8]	S[9]	S[10]
1	27.36	28.53	29.38	27.92	27.63	27.66	29.83	26.86	27.88	28.21
2	29.50	27.16	29.55	30.24	29.04	27.46	29.21	27.93	27.84	26.06
3	34.09	30.50	30.22	33.63	29.90	31.76	31.23	28.88	32.18	31.00
4	32.73	29.01	30.62	30.37	31.05	29.36	30.54	29.10	30.62	31.75
5	31.88	31.63	28.81	30.25	28.94	30.02	30.28	30.91	28.21	29.02
6	33.12	30.87	27.90	31.03	30.26	29.06	29.85	30.63	29.01	30.20
7	30.89	30.59	29.96	29.94	27.60	29.50	31.06	29.67	30.66	30.80
8	30.65	32.38	31.29	32.43	29.32	31.93	32.01	28.35	30.62	32.52
9	30.27	32.34	30.81	31.70	30.44	32.61	33.29	28.61	31.60	28.53
10	26.73	27.43	26.36	29.11	28.27	27.52	28.65	30.05	30.60	24.66
11	24.06	25.85	25.15	23.77	23.28	25.12	24.18	25.18	29.49	24.15
12	22.89	24.67	20.02	23.57	21.21	23.29	22.85	21.70	23.09	21.95
13	25.32	30.96	25.28	30.46	27.78	28.41	28.39	29.57	27.78	26.56
14	27.46	29.54	28.30	28.18	31.21	29.53	25.33	31.03	27.30	29.58
15	30.74	31.70	31.77	30.48	31.56	30.86	28.31	33.18	29.95	32.21
16	33.35	28.95	31.43	30.62	30.58	31.54	28.76	32.14	33.22	30.12
17	33.58	30.48	31.67	31.29	30.40	28.82	28.35	30.74	27.33	30.47
18	32.84	30.10	29.65	27.68	28.41	31.48	29.14	28.96	30.96	32.02
19	28.46	31.40	29.17	31.27	27.02	32.09	30.26	28.77	29.09	28.61
20	28.11	33.54	31.95	32.12	31.85	30.79	29.26	30.10	28.68	30.29
21	25.93	30.49	29.79	31.81	33.26	29.67	32.13	28.27	31.07	29.16
22	25.94	27.71	26.67	27.58	28.76	26.52	29.23	26.93	28.32	30.73
23	24.35	21.53	23.02	23.09	23.78	26.71	23.69	25.49	25.61	27.06
24	21.28	21.69	20.57	23.71	21.11	22.82	23.45	22.73	24.04	26.54
25	27.69	28.78	25.91	29.02	25.60	29.88	27.21	26.06	26.79	28.33
26	28.23	27.84	31.98	31.08	25.44	27.27	28.26	30.04	28.58	26.61
27	32.22	29.66	34.40	30.73	29.31	29.29	32.25	32.02	30.58	27.02
28	31.88	31.64	30.56	30.59	30.94	32.48	30.78	33.68	29.63	28.64
29	28.52	31.89	29.68	30.56	29.66	33.82	31.55	30.73	28.90	30.28
30	31.34	29.97	31.46	29.33	31.85	32.16	31.98	31.08	29.11	29.77
31	31.25	31.62	32.33	29.82	31.84	29.73	29.68	30.36	32.10	28.71
32	29.38	31.62	30.15	30.90	31.24	30.23	32.74	29.34	31.31	28.98
33	30.37	30.01	29.68	32.13	31.30	29.02	31.17	28.76	30.52	30.15
34	28.64	29.54	26.54	28.42	31.56	23.96	28.77	25.34	25.39	31.61
35	23.40	24.61	23.18	22.90	24.73	25.09	23.26	24.60	22.58	27.50
36	22.35	21.81	21.43	23.20	21.96	21.41	27.13	20.67	23.97	21.95

Fig. S2 illustrates a comparison between the forecasted water temperature values for three years and the original longitudinal data series using an ARIMAX model. The forecasted values display variations when compared to the original data series. Forecasts predict a significant rise in water temperature, increasing by 1.58 °C–7.12 °C from November 2024 to January 2025. This warming trend is expected to benefit the health and early developmental stages of tilapia broodfish. In contrast, a projected decline in water temperature ranging from 2.49 °C to 4.63 °C from February to March could raise concerns about the production and well-being of tilapia broodfish. During the central months of the year, the predicted temperatures reflect almost closer trends seen in the historical data. The comparative graphs indicate a noticeable seasonal pattern in water temperature changes. This pattern could potentially impact fish reproduction, both delaying and advancing broodfish reproduction. It is likely that exogenous climatic factors, such as air temperature and solar intensity, are contributing to these phenomena in the pond's water bodies.

## 4 Discussions

Understanding the influence of climatic variables on water quality in a Tilapia broodfish pond is crucial for ensuring the health and well-being of the fish. Factors like temperature, dissolved oxygen, pH, and nutrient levels significantly affect fish condition, reproductive success, and growth. By deciphering these connections, fish farmers can maintain optimal conditions, reduce stress, and minimize disease outbreaks. The environmental sustainability of a Tilapia broodfish pond relies on vigilant water quality management. Poor conditions can lead to algal blooms, oxygen depletion, and nutrient pollution, affecting the broader ecosystem. This knowledge is vital for economic success, regulatory compliance, and adapting to climate change. Understanding climatic impacts on water quality is essential for maintaining fish health, aquaculture viability, and environmental preservation. Limited studies have addressed these multifactorial issues, and forecasting pond water temperature with ARIMAX in this context has not been considered, making it valuable for stakeholders.

In our current study, we observed fluctuations in water quality parameters over the study period. Notably, water temperature reached its peak at 31.23 °C in October but dropped to 20.8 °C by December. It was evident that as air temperature increased, water temperature also rose. Furthermore, pH levels ranged from a high of 10.32 in August to a low of 7.36 in December. Similarly, dissolved oxygen levels peaked at 10.65 mg/L in January and declined to 7.09 mg/L in September, with ammonia levels spiking at 0.33 mg/L in February. Water transparency exhibited variations from 15.37 inches in June to 28 inches in January. Furthermore, we noted seasonal variations in climatic factors that could potentially impact water quality parameters, influencing their internal characteristics within the pond. These climatic variations can result in significant changes in water quality parameters. Our analysis exposed strong correlations between climatic variables and water quality parameters, with the first canonical function demonstrating a robust 89 % correlation. Air temperature (0.990) and wind speed (0.546) played a substantial role, explaining 27.2 % of the variance, while water temperature (0.937), pH (0.669), and dissolved oxygen (0.663) collectively accounted for 36.8 % of the variance. Notably, air temperature emerged as a particularly influential factor in driving variations in water temperature and subsequently affecting water quality parameters. The second, third, and fourth canonical functions also exhibited notable correlations of 43.1 %, 27.2 %, and 15.1 %, respectively, with water quality parameters.

Our study consistently aligns with the findings of several previous studies. Water temperature in Tilapia broodfish ponds is intricately linked to the ambient air temperature, rising with warmer air and decreasing when it's colder, directly affecting Tilapia's metabolism and behavior. There is a significant previous study emphasized the pivotal role of water temperature in the growth and survival of Nile tilapia fry, highlighting its crucial management in aquaculture settings [49]. Another study also pointed out the impact of fish ponds on nearby stream temperature patterns, with potential consequences for freshwater pearl mussel conservation [50]. A previous study further reported that highlighted the importance of water temperature in pond care, as it influences various factors such as water quality, metabolic rates, photosynthesis, oxygen levels, pH, and the biological activity and growth of aquatic life [51]. Understanding and effectively managing water temperature emerges as a critical aspect of successful aquaculture and pond maintenance [2]. Warmer air temperatures can decrease the solubility of oxygen in pond water, potentially resulting in lower dissolved oxygen (DO) levels [52,53]. Since, Tilapia requires adequate DO for respiration and growth, variations in DO can have a profound impact on their health and overall productivity [54,55]. It is reported that explains how temperature affects dissolved oxygen concentration in ponds [52]. The article states that temperature and dissolved oxygen have an inverse relationship, meaning that as temperature increases in water, DO levels decrease [[56], [57], [58], [59]]. The article also explains that cold water can hold more dissolved oxygen than warm water. According to another article reported by a previous study explains that aquatic animals are strongly affected by temperature, and aquaculture operations must be timed to correspond to water temperature [60]. Temperature is an important factor affecting the growth and survival of all organisms. The article also explains that warm water has a lower viscosity than cooler water, which favors settling rates of suspended particles and seepage of water through pond soils. A previous study discussed the influence of temperature on the respiratory rate of Nile Tilapia [61]. The study found that temperature has a significant effect on the oxygen consumption of Nile Tilapia, which is an important factor in aquaculture management. Studies like "Effects of temperature on oxygen consumption in Nile tilapia have investigated into the relationship between temperature and oxygen consumption in Tilapia, shedding light on the importance of managing DO in aquaculture systems [62]. Rainfall can be a significant climatic factor influencing pH levels in Tilapia broodfish ponds. Heavy rainfall can dilute the pond water, potentially leading to a decrease in pH due to the addition of acidic rainwater. Fluctuations in pH levels, as demonstrated in research on the "Effect of water hardness on the toxicity of acidic pH to rainbow trout (*Salmo gairdneri*)," can pose significant risks to the health and well-being of Tilapia within aquaculture systems [[63], [64], [65], [66]]. Solar intensity can have a dual impact on ammonia concentrations in Tilapia broodfish ponds. On one hand, solar radiation can enhance photosynthesis in phytoplankton, leading to a reduction in ammonia levels as phytoplankton utilize ammonia for growth. However, excessive solar intensity without proper nutrient management can lead to the proliferation of algae and harmful algal blooms, potentially increasing ammonia levels to detrimental levels [67]. This study found the temporal variation of ammonia and nitrite content in extensive tilapia ponds [67]. The study found that the mean value of temperature in extensive tilapia ponds was 28.5 °C, while the mean value of pH was 7.5. The mean value of dissolved oxygen was 5.5 mg/L, while the mean value of total ammonia nitrogen was 0.15 mg/L.A study investigated the impact of water de-stratification on dissolved oxygen and ammonia levels in tilapia ponds located in Northern Thailan [68]. The study observed that thermal de-stratification typically occurred late at night during the hot and dry seasons, while in the wet season, it happened earlier in the evening due to the cooling effect of rain. The mixing of surface and bottom waters led to a decrease in dissolved oxygen near the surface and an increase in its concentration in the deeper layers. In 68 % of the observations, mean dissolved oxygen (DO) levels in integrated and commercial ponds were below 1 mg/L between 02:00 and 06:00 h. The repeated measures ANOVA revealed that water depth and fish farming systems significantly influenced total ammonia nitrogen (TAN). TAN levels were lower near the surface compared to the bottom and increased following water de-stratification.Research, such as "Effect of light on the inhibition of ammonia oxidation by nitrapyrin in soil and aquatic environments," was conducted previously and yielded significant findings about the impact of light [69]. One previous study has investigated the intricate relationships between light availability and ammonia dynamics in aquatic environments [70,71].Humidity levels and solar intensity play crucial roles in determining water transparency in Tilapia broodfish ponds. High humidity and intense sunlight can promote the growth of algae and suspended particles in the water, leading to reduced water transparency [72,73]. This reduction in water transparency can adversely affect the feeding efficiency and behavior of Tilapia, potentially impacting their growth and overall health [[74], [75], [76]]. Scientific studies like influence of light availability on the spatial distribution and feeding behavior of two cyprinid species have explored how light conditions in aquatic environments can influence the behavior and distribution of fish species, offering valuable insights into the importance of water transparency in aquaculture management [77,78].

The ARIMA model, renowned for its effectiveness and dynamic application in analyzing time series data and making forecasts, has been utilized in numerous prior research studies [53,[79], [80], [81]]. This same model has been adopted in studies with a similar focus on its efficacy, as reported by several studies [[79], [80], [81]]. Further validation of the suitability of ARIMA modeling was carried out by this study, particularly in forecasting fish production [80]. In a different context, a researcher evaluated the accuracy of their ARIMA model in forecasting daily confirmed COVID-19 cases in India [80].Additional research efforts, like the study conducted by a renowned researcher have harnessed ARIMA modeling to predict fish production in diverse regions, including Tamil Nadu, Assam, Odisha, and the Chilika lagoon in India [46]. To assess the forecasting capacity of the ARIMA model fitted for our research, we employed a crucial metric for evaluating prediction accuracy during the study period, following the approach outlined by a previous study [82]. In addressing the complexity of fluctuating water temperatures influenced by external factors, we introduced the ARIMAX model, a versatile extension of ARIMA that considers exogenous variables. The choice of ARIMAX proved to be suitable for forecasting water temperature in broodfish ponds, especially given the significant impact of external factors like air temperature and solar intensity on the water's internal quality parameters. Importantly, our analysis determined that the observed water temperature data exhibited stationarity and was notably free from noise. This study underlined the significant influence of certain elements on evaluation accuracy and result adequacy. In response, we employed a method involving smoothing the time series longitudinal data to simplify the process and mitigate the effects of noise components.

We applied the ARIMA (p, d, q) technique to analyze the longitudinal time series data of water temperature. Comprehensive testing, including the Augmented Dickey-Fuller (ADF) test (yielding p-values below 0.05) and the normal probability distribution plot test (with values above 0.05), verified that the pond water temperature data was stationary. The inherent stationarity of the data was further demonstrated by a rapid decrease in correlations observed in the Autocorrelation Function (ACF) and Partial Autocorrelation Function (PACF) plots. In the ACF, we observed a diminishing association with increasing time lags, while in the PACF, we noticed prominent spikes at the initial time lags. These observations collectively indicated that the data was inherently stationary, without persistent trends or seasonality, making it amenable to straightforward time series analysis without requiring complex transformations. The model's suitability was corroborated by the lowest normalized Bayesian Information Criterion (BIC) values and the visual patterns in ACF and PACF plots, as similarly reported by several studies [80,[83], [84], [85], [86], [87], [88]].

Before implementing ARIMAX for forecasting, we initially developed an ARIMA model. Notably, the AR and MA values of the ARIMA model were the same as those in the ARIMAX model. The best-fitting model for tilapia production was identified as ARIMAX (1, 0, 1). Similarly, the most suitable model for water temperature was determined to be ARIMAX (1, 0, 1), supported by the lowest normalized BIC values and the visual patterns in ACF and PACF plots. The analysis conducted in this study affirmed that the optimal model for forecasting water temperature was ARIMAX (1, 0, 1), based on the values obtained for Root Mean Square Error (RMSE), Mean Absolute Percentage Error (MAPE), Maximum Absolute Percentage Error (MaxAPE), Mean Absolute Error (MAE), Maximum Absolute Error (MaxAE), and Normalized BIC, in addition to the normal distribution of residual ACF and PACF spikes. Previous studies have underscored the significance of employing appropriate time series models to accurately depict observed data and generate dependable projections for pond water temperature [85,[88], [89], [90]]. There are few studies found about the modeling and forecasting with ARIMAXand but the application of this model in particular case fisheries is still unavailable [84,[91], [92], [93], [94]].

We explored the critical relationship between climate-related factors and water quality parameters, focusing on their impact on water quality in tilapia broodfish ponds. To predict future water temperature trends, we employed an ARIMAX model. Our analysis revealed that the most accurate model was ARIMA (1, 0, 1), which demonstrated a fluctuating pattern influenced by air temperature from February 2022 to January 2025, with a slight overall increase. If this pattern persists, we anticipate the water temperature in the tilapia broodfish pond will reach 27.93 °C by the end of January 2025, surpassing the temperature observed in January 2022. Through simulation, we observed a distinct seasonal pattern in the water temperature of the tilapia broodfish pond. Temperatures gradually increased from around 21 °C in December and January to a peak of approximately 34 °C in July, August, and September, followed by a gradual decline in autumn and winter. This cyclical temperature variation was a response to external factors, primarily air temperature and solar intensity. These findings held practical significance for our pond management and contributed valuable information for our ecological conservation efforts, deepening our understanding of how seasonal temperature changes impact local aquatic ecosystems and habitats. Comparing three-year forecasted values of water temperatures using an ARIMAX model to the original data, variations were evident. Remarkably, the forecasts have suggested a cooling trend during February to March, which could potentially negative impact the development and growth of broodfish. Conversely, a warmer temperature is expected from November to January, which is likely to be beneficial for the growth and overall development of broodfish. These temperature changes could impact fish reproduction, likely influenced by external factors like air temperature and solar intensity. These insights provide valuable information to a wide range of stakeholders, including researchers, policymakers, educators, and individuals involved in tilapia farming. They can use this information to enhance their ability to forecast future pond conditions, taking into account external factors such as air temperature. As a result, these findings hold substantial significance for those seeking to develop effective strategies and policies for sustainable broodfish pond management, both within Bangladesh and in similar global environments.

We acknowledge the limitations inherent in modeling and predicting water temperature using ARIMAX with longitudinal time series data. Our approach primarily focused on statistical modeling and its implications for water quality to forecast future scenarios. While we considered external influential factors, specifically air temperature and solar intensity, on pond water quality, it's important to recognize that our analysis did not encompass all potential influencing factors in the pond system that could significantly affect water temperature. Obtaining extensive time series data for robust modeling and forecasting presented challenges, particularly for conducting a long-term water quality study over an extended period in a specific location.

## 5 Conclusion

In conclusion, climate change can significantly impact aquatic ecosystems by altering their physical and chemical characteristics. This study evaluated the effects of climatic factors on water quality parameters and predicted water temperature in a tilapia broodfish pond using an ARIMAX model. It was evident that climatic factors played a substantial role in determining water quality parameters throughout the study period. The ARIMAX (1, 0, 1) model, with air temperature and solar intensity as key exogenous influential factors, proved to be the best-fit model, projecting a trend of increasing water temperature. If this pattern persists, the water temperature in the pond is projected to increase from November to January, outstanding previous records, and this is expected to be beneficial for the growth and overall development of broodfish. But it is noteworthy that the forecasts also predict a cooling trend and a decline in water temperature from February to March, which could adversely affect broodfish growth and development during this time. There is a fluctuating and seasonal pattern of water temperature expected in the future. However, the ARIMAX model considered only air temperature and solar intensity as exogenous factors, excluding other potential influences in the pond ecosystem. Future studies should incorporate a broader range of factors for a more comprehensive analysis. Therefore, the present findings provide valuable insights for a range of stakeholders, including researchers, policymakers, educators, and tilapia farming entrepreneurs, offering them the means to better prepare for future pond management by considering the impact of climatic factors and water quality parameters in their planning and decision-making processes.

## Ethics declaration

This study involved various activities, one of which was collecting water samples for assessing water quality parameters. The sample collection process was conducted in a manner that ensured the well-being of other aquatic animals and did not disrupt the aquatic environment. These activities adhered to strict animal research guidelines, and the necessary approval was obtained from the Animal Welfare and Ethics Committee at Bangladesh Agricultural University (BAU), with reference code BAURES/ESRC/FISH-11/2022. The use of climatic factors data complies with the terms of use from the original sources, including the Bangladesh Meteorological Department, with appropriate citations (https://live6.bmd.gov.bd/). There was no unauthorized access or alteration of data in this study. The research adheres to ethical guidelines, safeguarding individual confidentiality and privacy while promoting responsible conduct in research.

## Data availability statement

The data presented in this study are available upon request from the corresponding author.

## CRediT authorship contribution statement

**Mohammad Abu Baker Siddique:** Writing – original draft, Visualization, Validation, Software, Methodology, Formal analysis, Data curation. **Balaram Mahalder:** Writing – review & editing, Validation, Data curation. **Mohammad Mahfujul Haque:** Writing – review & editing, Validation, Supervision, Conceptualization. **A. K. Shakur Ahammad:** Writing – review & editing, Visualization, Supervision, Resources, Project administration, Methodology, Investigation, Funding acquisition, Conceptualization.

## Declaration of competing interest

The authors declare that they have no known competing financial interests or personal relationships that could have appeared to influence the work reported in this paper.
