# Supplementary material for: Impact of climatic factors on water quality parameters in tilapia broodfish ponds and predictive modeling of pond water temperature with ARIMAX
Source: Heliyon. 2024 Sep 11;10(18):e37717. doi: 10.1016/j.heliyon.2024.e37717 (PMC11422597; doi:10.1016/j.heliyon.2024.e37717)
Supplement: Multimedia component 1 [file mmc1.docx]

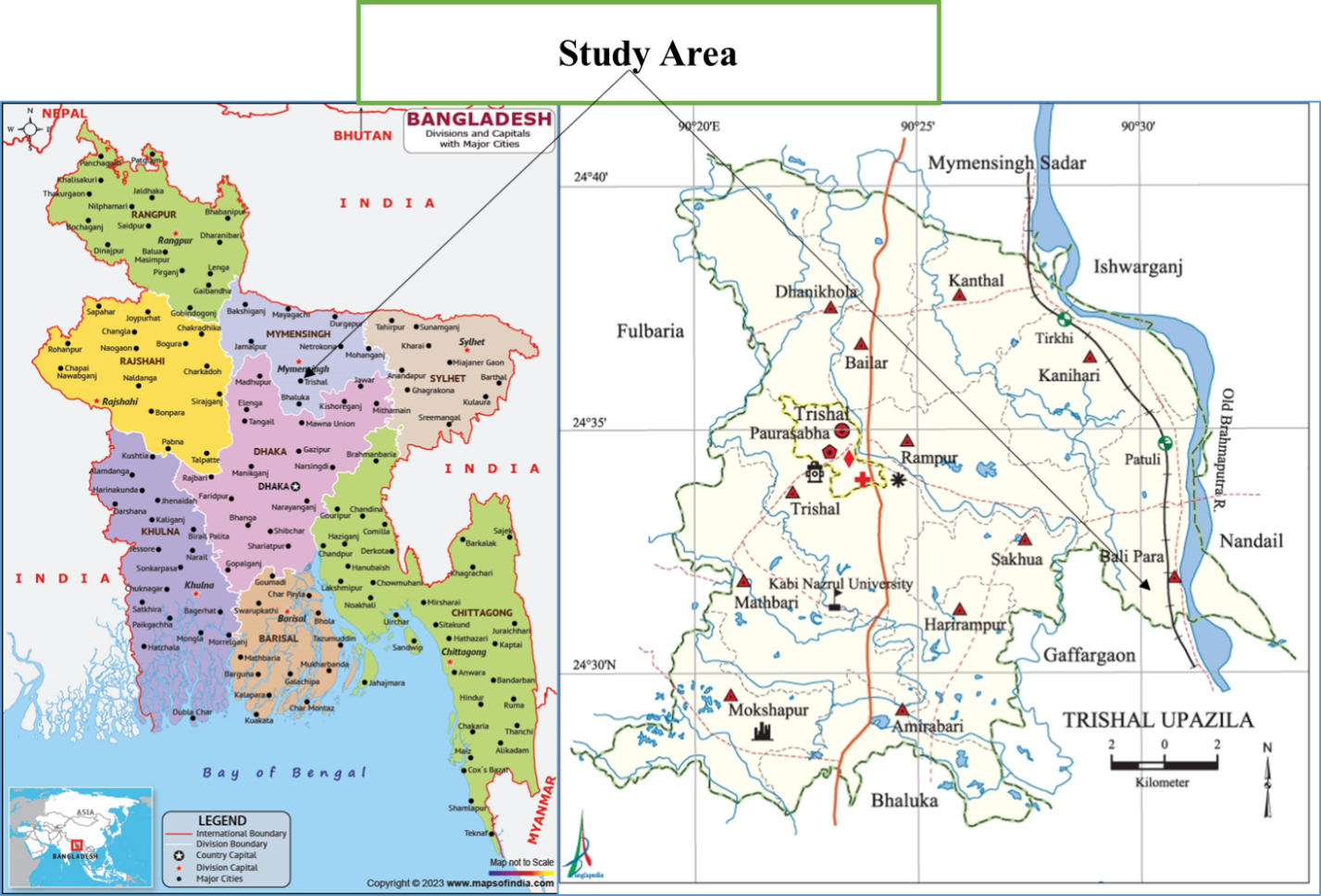


Figure S1: Study area: Dhala, Trishal, Mymensingh

**
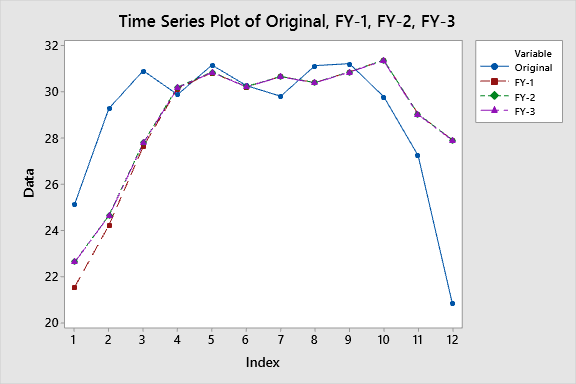
**

Figure S2. A comparison of three years forecasting values of water temperature with original data series using ARIMAX
